# Assessing the Stability and Safety of Procedure during Endoscopic Submucosal Dissection According to Sedation Methods: A Randomized Trial

**DOI:** 10.1371/journal.pone.0120529

**Published:** 2015-03-24

**Authors:** Chan Hyuk Park, Seokyung Shin, Sang Kil Lee, Hyuk Lee, Yong Chan Lee, Jun Chul Park, Young Chul Yoo

**Affiliations:** 1 Division of Gastroenterology, Department of Internal Medicine, Severance Hospital, Institute of Gastroenterology, Yonsei University College of Medicine, Seoul, Korea; 2 Department of Anesthesiology and Pain Medicine, Severance Hospital, Anesthesia and Pain Research Institute, Yonsei University College of Medicine, Seoul, Korea; 3 Division of Gastroenterology, Department of Medicine, Samsung Medical Center, Sungkyunkwan University School of Medicine, Seoul, Korea

## Abstract

**Background:**

Although endoscopic submucosal dissection (ESD) is routinely performed under sedation, the difference in ESD performance according to sedation method is not well known. This study attempted to prospectively assess and compare the satisfaction of the endoscopists and patient stability during ESD between two sedation methods.

**Methods:**

One hundred and fifty-four adult patients scheduled for ESD were sedated by either the IMIE (intermittent midazolam/propofol injection by endoscopist) or CPIA (continuous propofol infusion by anesthesiologist) method. The primary endpoint of this study was to compare the level of satisfaction of the endoscopists between the two groups. The secondary endpoints included level of satisfaction of the patients, patient’s pain scores, events interfering with the procedure, incidence of unintended deep sedation, hemodynamic and respiratory events, and ESD outcomes and complications.

**Results:**

Level of satisfaction of the endoscopists was significantly higher in the CPIA Group compared to the IMIE group (IMIE vs. CPIA; high satisfaction score; 63.2% vs. 87.2%, *P*=0.001). The incidence of unintended deep sedation was significantly higher in the IMIE Group compared to the CPIA Group (IMIE vs. CPIA; 17.1% vs. 5.1%, *P*=0.018) as well as the number of patients showing spontaneous movement or those requiring physical restraint (IMIE vs. CPIA; spontaneous movement; 60.5% vs. 42.3%, *P*=0.024, physical restraint; 27.6% vs. 10.3%, *P*=0.006, respectively). In contrast, level of satisfaction of the patients were found to be significantly higher in the IMIE Group (IMIE vs. CPIA; high satisfaction score; 85.5% vs. 67.9%, *P*=0.027). Pain scores of the patients, hemodynamic and respiratory events, and ESD outcomes and complications were not different between the two groups.

**Conclusion:**

Continuous propofol and remifentanil infusion by an anesthesiologist during ESD can increase the satisfaction levels of the endoscopists by providing a more stable state of sedation.

**Trial Registration:**

ClinicalTrials.gov NCT01806753

## Introduction

Since it was first developed in the late 1990s, endoscopic submucosal dissection (ESD) has gained much popularity and has been proven to be superior to endoscopic mucosal resection (EMR) by showing higher rates of en-bloc resection in larger tumors and in expanded indications for endoscopic resection of early gastric cancer [1]. However, despite the continuous development in endoscopic devices, ESD is still a technically challenging procedure and usually takes longer than EMR [1–3]. The risk of complications such as perforation [4] and bleeding [5] is known to increase with such lengthy procedures, as well as patient discomfort and pain. Minimizing patient movement can therefore be said to be of key importance for a successful procedure, and it has been suggested by many that deep sedation is preferable during ESD in order to enhance the safety and precision of the procedure [3,6]. However, methods of propofol-based moderate sedation have been recently found to be a safe and effective option during therapeutic gastrointestinal (GI) endoscopic procedures by providing better patient cooperation [7,8].

In terms of sedative agents, propofol has greatly replaced the role of benzodiazepines during the past decade, and better results with propofol sedation during ESD have been reported in several studies [9–11]. The main problems with propofol, however, were its narrow therapeutic window, lack of reversal agent and considerable risk of respiratory depression [12]. This has resulted in many studies aimed to resolve the issue of ‘who’ should administrate propofol and ‘how’ it should be done with regard to safety, cost and legal aspects [13–16]. On the other hand, the relationship between sedation methods and performance of ESD has not been widely studied. A recent retrospective analysis found the sedation method to significantly affect clinical outcomes of ESD [17]. More specifically, this study concluded that continuous propofol infusion supplemented with opioid administration by anesthesiologists improved ESD performance by increasing en bloc resection and complete resection rates and reducing procedure time, compared to intermittent bolus injection of midazolam and propofol by endoscopists. These results were largely attributed to the enhanced stability of the procedure by a decrease in patient movement and other events that may interrupt a smooth intervention, and a general increase in the satisfaction of the endoscopists was also assumed with continuous propofol infusion used by the anesthesiologists. However, this study failed to present satisfaction scores of the endoscopists or the patients, and had the limitation of being a retrospective analysis. We therefore aimed to prospectively assess the satisfaction of the endoscopists as well as patient stability during ESD procedures according to sedation method in the present study.

## Patients and Methods

The protocol for this trial and supporting CONSORT checklist are available as supporting information; see S1 Checklist and S1 Protocol.

### Study population

The study protocol was approved by the Institutional Review Board and Hospital Research Ethics Committee of Severance Hospital, and registered at http://clinicaltrials.gov (registration number NCT01806753). Adult patients between the age of 20 to 80 years that were diagnosed with early gastric cancer or adenoma and scheduled for ESD were recruited for this study. All patients were of ECOG performance status 0 or 1 and American Society of Anesthesiologists (ASA) physical status I~III. Written informed consent was obtained from all patients. Patients that had previously undergone subtotal gastrectomy or gastrostomy, those receiving repeated ESD or presenting with three or more synchronous lesions were excluded from this study. Pregnant or breastfeeding patients and those with known allergies to eggs, soy beans or sulfites were excluded from this study. Patients that had received sedation for another procedure within 24 hours prior to ESD, those with debilitating neurologic or psychotic disorders or unable to provide informed consent were also excluded.

### Study design, sedation protocols and method of ESD

Enrolled patients were randomly assigned in a 1:1 ratio by using a table of computer-generated random numbers with sealed envelopes to either the IMIE (intermittent midazolam/propofol injection by endoscopist) Group or the CPIA (continuous propofol infusion by anesthesiologist) Group. This was created by one of the investigators (CHP) by applying a block randomization method with a block size of four. Although the patients remained blinded to group assignment until discharge, endoscopists inevitably became aware of the sedation method used in each patient while performing ESD.

The targeted level of sedation was Modified Observer’s Assessment of Alertness/Sedation (MOAA/S) score of 3 or 4 in both groups (Table 1) [18]. Sedation in the IMIE Group was done by endoscopists who were not involved in the endoscopic procedure. All three endoscopists performing sedation were familiar with procedural sedation, and were fully informed of the sedation protocols of the present study. Sedation was started by administrating an initial bolus of 50 mg of meperidine intramuscularly, and 0.05 mg/kg of intravenous (IV) midazolam. When patients were found to be undersedated with an MOAA/S score of 5 or 6, bolus doses of 0.25 mg/kg of propofol were given to maintain the targeted depth of sedation. Patients showing signs of discomfort or pain such as spontaneous movements while presenting with an MOAA/S score of 3 or 4 were assessed as inappropriate analgesia, and were given additional bolus doses of meperidine 12.5 mg intravenously. Sedation in the CPIA Group was performed by a three qualified anesthesiology staff members trained for procedural sedation outside the operating theater. Initial bolus doses of 0.5 μg/kg of remifentanil and 0.5 mg/kg of propofol were followed by continuous infusions of remifentanil and propofol at 0.08 μg/kg/min and 2 mg/kg/hr, respectively by using an automated pump. Undersedated patients presenting with an MOAA/S score of 5 or 6 were given additional bolus doses of 0.25 mg/kg propofol and the infusion rate was increased by 0.5 mg/kg/hr. Patients of MOAA/S score 3 or 4 and showing signs of discomfort or pain were given additional analgesia by increasing the infusion rate of remifentanil by 0.02 μg/kg/min. When the mean blood pressure of the patient fell below 60 mmHg or decreased by more than 20% from baseline, or the patient showed desaturation of SpO_2_ <90%, propofol infusion rates were decreased by 0.5 mg/kg/hr.

**Table 1:** Modified Observer’s Assessment of Alertness/Sedation Scale [18].

Responsiveness	Score
Agitated	6
Responds readily to name spoken in normal tone (alert)	5
Lethargic response to name spoken in normal tone	4
Responds only after name is called loudly and/or repeatedly	3
Responds only after mild prodding or shaking	2
Does not respond to mild prodding or shaking	1
Does not respond to deep stimulus	0

Four attending gastroenterologists performed the ESD procedures in this study. All ESD procedures were performed with a standard single-channel endoscope. The typical procedure sequence consisted of marking, mucosal incision, and submucosal dissection with simultaneous hemostasis. After making several marking dots circumferentially around the lesion with a needle knife or a needle knife papillotome, a saline solution containing epinephrine (0.01 mg/mL) mixed with 0.8% indigo carmine was injected into the submucosal layer using a 21-gauge needle to lift the lesion away from the muscle layer. A circumferential incision was made in the mucosa using a needle knife and an insulated-tip knife. The submucosal layer was dissected directly with various knives until complete removal was achieved. Endoscopic hemostasis was performed with hemoclips or hemostatic forceps whenever bleeding or exposed vessels were observed.

### Patient monitoring

All ESD procedures were performed in a room dedicated to upper gastrointestinal endoscopy and fully equipped for advanced cardiac life support. Patients were to arrive at the endoscopy room with secure IV access, and were administered normal saline or Hartmann solution as appropriate. Nasal oxygen was supplied at 2 L/min. Patient monitoring included noninvasive blood pressure measurements every 5 minutes, pulse oximetry (SpO_2_), electrocardiography and respiratory activity via thoracic leads. Depth of sedation was assessed by the MOAA/S score at four time points; just before the insertion of the endoscope, after insertion of the endoscope and before the first incision, immediately after the first incision and at the end of dissection. Additional assessments of sedation depth were done when patients showed signs of undersedation or reactions to discomfort and/or pain. At the end of ESD, patients were transferred to the endoscopy recovery unit and monitored by noninvasive blood pressure, pulse oximetry and electrocardiography. Assessment of recovery was done by dedicated nursing staff by using the modified Aldrete score every 5 minutes until discharge [19].

### Outcome measures

Before the procedure, demographic data of the patients including age, sex, height, weight, smoking history, underlying diseases, and medication history were obtained. In addition, tumor location and macroscopic types were endoscopically evaluated and classified according to the Japanese Gastric Cancer Association Classification [20]. Tumor size, presence of ulceration, invasion depth, lymphatic and vascular involvement, and tumor involvement at the lateral and vertical margins were histopathologically assessed. During the procedure, the following sedation-related events were observed and collected: episodes of unintended deep sedation, events intefering with the procedure (belching, vomiting, spontaneous movements and the need for physical restraint), hemodynamic events (hypertension, hypotension, tachycardia, and bradycardia), and respiratory events (chin lift/jaw thrust maneuver, increase in oxygen flow, and assisted mask ventilation). The endoscopists gave their satisfaction scores at the end of the procedure on a verbally administered numerical rating scale of 0 to 10, which were later categorized as satisfaction levels of either low (0–3), medium (4–6), or high (7–10), for analysis. Satisfaction scores of the patients were assessed and categorized in the same manner as the endoscopists at the following morning after the procedure. Pain scores of the patients were assessed after full recovery at the endoscopy recovery unit and at the following morning after the procedure on a visual analogue scale of 0 to 10, 0 meaning no pain and 10 meaning worst pain imaginable. Finally, ESD outcomes including en-bloc resection, complete resection, and curative resection and ESD complications including post-procedural bleeding, peforation, and aspiration pneumonia were assessed.

### Definitions

Procedure time was defined as time from marking to complete removal, including the time required for hemostasis. MOAA/S scores lower than 3 were defined as unintended deep sedation, and events interfering with the procedure were noted as belching, vomiting, spontaneous movement or uncooperative patients requiring physical restraint. Respiratory events were defined as apnea or desaturation (SpO_2_ < 90%) that required either a chin lift or jaw thrust maneuver, an increase in O_2_ flow or assisted mask ventilation. Hypertension was defined as a greater than 20% increase in mean blood pressure from baseline. Hypotension was defined as when the mean blood pressure of the patient fell below 60 mmHg or decreased by more than 20% from baseline. Tachycardia and bradycardia were each defined as a heart rate >120 beats/min and <50 beats/min, respectively. Full recovery of the patients was defined as an Aldrete score of 10.

En bloc resection was defined as the resection of a single piece, as opposed to the resection of multiple pieces. Complete resection was defined as tumor-free lateral and vertical margins on histologic examination. Curative resection was defined as an en bloc resected lesion with negative margins of neoplasm and which fulfilled the criteria of node-negative neoplasms with no lymphovascular infiltration.

### Study endpoints

The primary endpoint of this study was to compare the level of satisfaction of the endoscopists between the two groups. In addition, the following secondary endpoints were assessed: level of satisfaction of the patients, patient’s pain scores, events interfering with the procedure, incidence of unintended deep sedation, hemodynamic and respiratory events, outcomes of ESD including en-bloc, complete, and curative resection, and complications of ESD including post-procedural bleeding, perforation, and aspiration pneumonia.

### Statistical analysis

Sample size calculation was performed using a χ^2^ test based on the results of our pilot study. Number of patients according to the satisfaction level was as follows: one with low satisfaction scores (0–3), one with medium satisfaction scores (4–6), and eight with high satisfaction scores (7–10) under the CPIA method, and one with low satisfaction scores (0–3), three with medium satisfaction scores (4–6), and six with high satisfaction scores (7–10) under the IMIE method. Based on the results of the pilot study, the effect size was estimated as 0.25. In addition, the degree of freedom was two because values of the primary outcome were classified into a 2 x 3 contingency table. As a result, a sample size of 155 would be needed to achieve 80% power to detect an effect size of 0.25 using a χ^2^ test with 2 degrees of freedom, with a significance level of 0.05. We recruited 157 patients considering a 1% dropout rate. Continuous variables with normal distribution were analyzed with the independent two sample *t*-test. Categorical variables were analyzed by the χ^2^ or Fisher’s exact test. In order to adjust possible confounding variables, the primary outcome was further analyzed by logistic regression analysis. All statistical analyses were performed with IBM SPSS Statistics 20.0 (IBM Corp., Armonk, NY, USA). A *P* value of <0.05 was considered statistically significant.

## Results

**Fig 1:**
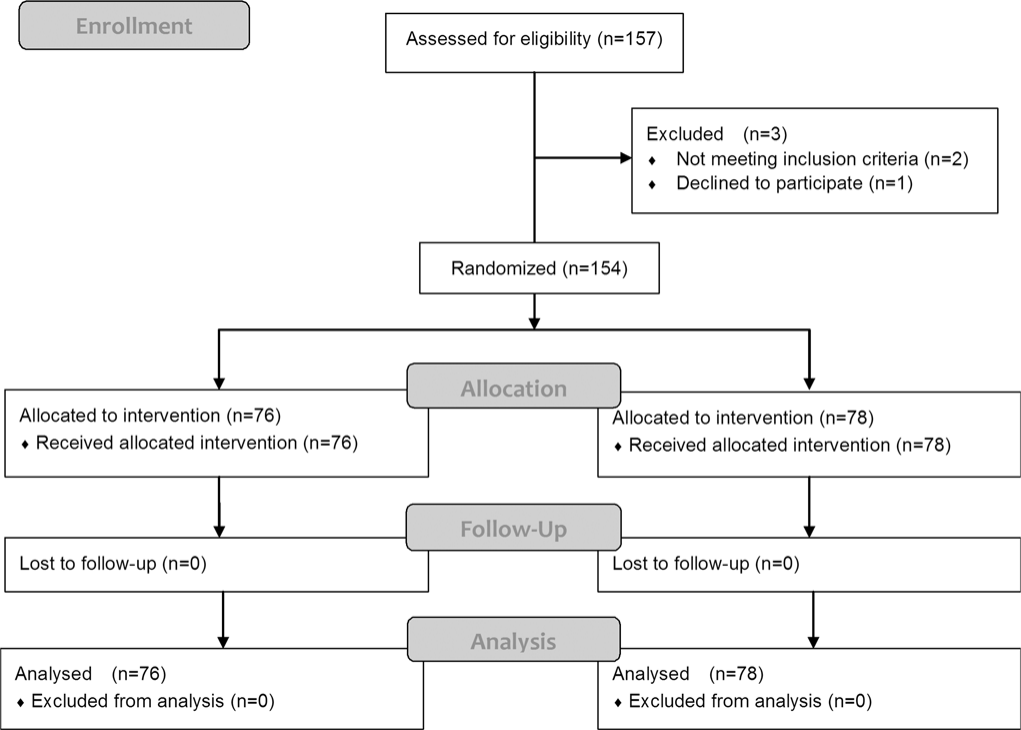
CONSORT flow chart of patient sample selection.

The CONSORT flow diagram of this study is shown in Fig. 1. Among the 157 patients that were assessed for eligibility between March and December 2013, 2 patients that underwent endoscopic ultrasound under sedation just before ESD and 1 patient that declined to participate were excluded from the study. The remaining 154 patients were randomly assigned to either the IMIE Group (n = 76) or the CPIA Group (n = 78). After randomization, all patients received their allocated intervention and none were lost to follow-up or excluded from analysis.

### Demographic data

Demographic data are shown in Table 2. There were no differences in patient age, sex, body mass index, smoking history, ASA physical status distribution or the history of the use of antiplatelet agents or anticoagulants between the two groups. Antiplatelet agents and anticoagulants were discontinued in all patients prior to endoscopic submucosal dissection.

**Table 2:** Demographic data.

**Variable**	**IMIE**	**CPIA**	***P* value**
	**(n = 76)**	**(n = 78)**	
Age (years)	63.3 ± 8.8	61.0 ± 9.5	0.125
Sex, n (%)			0.442
Male	55 (72.4)	52 (66.7)	
Female	21 (27.6)	26 (33.3)	
Body mass index (kg/m^2^)	23.6 ± 3.2	24.2 ± 2.2	0.187
Smoking history, n (%)			0.381
Smoker	23 (30.3)	17 (21.8)	
Ex-smoker	20 (26.3)	27 (34.6)	
Non-smoker	33 (43.4)	34 (43.6)	
ASA physical status, n (%)			0.127
1	51 (67.1)	43 (55.1)	
2	21 (27.6)	24 (30.8)	
3	4 (5.3)	11 (14.1)	
Use of antiplatelet agents or anticoagulants,^a^ n (%)	4 (5.3)	10 (12.8)	0.103

Values are mean ± SD or n (%) of patients.

^a^Antiplatelet agents or anticoagulants include aspirin, non-steroidal anti-inflammatory drugs and warfarin. The aforementioned drugs were discontinued in all patients prior to endoscopic submucosal dissection. ‘Use of antiplatelet agents or anticoagulants’ indicates the number of patients who took these medications.

IMIE, intermittent midazolam/propofol injection by endoscopist; CPIA, continuous propofol infusion by anesthesiologist; ASA, American Society of Anesthesiologists.

### Lesion characteristics and outcomes of endoscopic submucosal dissection

The numbers of lesions were 78 and 82 in the IMIE and CPIA Group, respectively. There was no difference between the two groups with regard to the location of lesions, macroscopic appearance, histology or size of the lesions. Ulceration was not found in any of the lesions of both groups. Depth of invasion or the incidence of lymphovascular invasion was also comparable between groups, as well as the outcomes and complications of ESD (Table 3).

**Table 3:** Lesion characteristics and outcomes of endoscopic submucosal dissection.

**Variable**	**IMIE**	**CPIA**	***P* value**
Number of lesions	78	82	
Location of lesions, n (%)			0.981
Upper third	6 (7.7)	7 (8.5)	
Middle third	28 (35.9)	29 (35.4)	
Lower third	44 (56.4)	46 (56.1)	
Macroscopic appearance, n (%)			0.675
Elevated	51 (65.4)	51 (62.2)	
Flat or depressed	27 (34.6)	31 (37.8)	
Presence of ulcer, n (%)	0 (0.0)	0 (0.0)	N/A
Histology, n (%)			0.661
Adenoma	46 (59.0)	44 (53.7)	
Differentiated cancer	29 (37.2)	36 (43.9)	
Undifferentiated cancer	3 (3.8)	2 (2.4)	
Lesion size, n (%)			0.224
≤ 10 mm	43 (55.1)	34 (41.5)	
10 to 20 mm	24 (30.8)	33 (40.2)	
> 20 mm	11 (14.1)	15 (18.3)	
Depth of invasion,^a^ n (%)			0.617
Mucosa	25 (78.1)	31 (81.6)	
Submucosa (< 500 μm)	5 (15.6)	3 (7.9)	
Submucosa (≥ 500 μm)	2 (6.3)	4 (10.5)	
Lymphovascular invasion, ^a^ n (%)	3 (9.4)	4 (10.5)	> 0.999
Outcomes of ESD, n (%)			
En-bloc resection	76 (97.4)	82 (100.0)	0.236
Complete resection	75 (96.2)	81 (98.8)	0.358
Curative resection	69 (88.5)	76 (92.7)	0.360
Complications of ESD, n (%)			
Post-procedural bleeding	2 (2.6)	3 (3.7)	> 0.999
Perforation	0 (0.0)	1 (1.2)	> 0.999
Pneumonia^b^	0 (0.0)	0 (0.0)	N/A

^a^The percentage of this variable was calculated based on the number of early gastric cancer lesions.

^b^The percentage of this variable was calculated based on the number of patients.

IMIE, intermittent midazolam/propofol injection by endoscopist; CPIA, continuous propofol infusion by anesthesiologist; ESD, endoscopic submucosal dissection; N/A, not applicable.

### Sedation-related data, speed of recovery and satisfaction scores

Table 4 shows data relevant to sedation, recovery, and satisfaction scores of the endoscopists and patients in all of the procedures. Procedure time was not significantly different between the two groups. The proportion of patients that reached complete recovery within 5 minutes of admission to the endoscopy recovery unit was significantly greater in the CPIA Group compared to the IMIE Group (*P* = 0.001). All patients in both groups were able to recover fully within 10 minutes.

**Table 4:** Sedation-related data, speed of recovery and satisfaction scores.

**Variable**	**IMIE**	**CPIA**	***P* value**
	**(n = 76)**	**(n = 78)**	
Procedure time, min	33.3 ± 21.8	38.0 ± 24.1	0.207
Drug requirements for sedation			
Midazolam, mg/kg	0.06 ± 0.01		
Pethidine, μg/kg/min	35.2 ± 17.4		
Propofol, μg/kg/min	46.6 ± 26.2	63.2 ± 33.1	
Remifentanil, μg/kg/hr		6.7 ± 3.7	
Speed of recovery, n (%)			
Within 5 minutes	57 (75.0)	74 (94.9)	0.001
Within 10 minutes	76 (100.0)	78 (100.0)	N/A
Level of satisfaction of the endoscopists, n (%)			0.001
Low	6 (7.9)	1 (1.3)	
Medium	22 (28.9)	9 (11.5)	
High	48 (63.2)	68 (87.2)	
Level of satisfaction of the patients, n (%)			0.027
Low	2 (2.6)	8 (10.3)	
Medium	9 (11.8)	17 (21.8)	
High	65 (85.5)	53 (67.9)	
Patient's pain score			
Immediately after the procedure	0.8 ± 1.5	1.1 ± 1.6	0.281
At the following morning after the procedure	2.8 ± 2.2	3.0 ± 1.7	0.374

Values are mean ± SD or n (%) of patients.

IMIE, intermittent midazolam/propofol injection by endoscopist; CPIA, continuous propofol infusion by anesthesiologist; N/A, not applicable.

Level of satisfaction of the endoscopists was found to be significantly different between the two groups, with the CPIA Group showing a higher degree of satisfaction (*P* = 0.001). In contrast, level of satisfaction of the patients were found to be significantly higher in the IMIE Group compared to the CPIA Group (*P* = 0.027). Pain scores reported by the patients were not different between the two groups both immediately after and at the following morning after the procedure. The logistic regression model shown in Table 5 confirmed that the CPIA group was associated with higher satisfaction scores under adjusting for possible confounding variables (odds ratio [95% confidence interval] = 4.217 [1.840–9.668]).

**Table 5:** Associate factor for affecting high satisfaction score of endoscopist.

**Variable**	**n**	**High satisfaction score**	**OR (95% CI)**	***P* value**
		**n (%)**		
Number of lesions				
One	148	112 (75.7)	1	
Two	6	4 (66.7)	0.541 (0.083–3.512)	0.519
Histology				
Adenoma	84	62 (73.8)	1	
Cancer	70	54 (77.1)	1.151 (0.513–2.584)	0.733
Location				
Upper or middle third	70	53 (75.7)	1.089 (0.484–2.447)	0.837
Lower third	84	63 (75.0)	1	
Procedure time, min	N/A	N/A	0.992 (0.975–1.009)	0.365
Sedation method				
IMIE	76	48 (63.2)	1	
CPIA	78	68 (87.2)	4.217 (1.840–9.668)	0.001

IMIE, intermittent midazolam/propofol injection by endoscopist; CPIA, continuous propofol infusion by anesthesiologist; OR, odds ratio; CI, confidence interval; N/A, not applicable.

### Adverse events

Adverse events were shown in Table 6. The incidence of unintended deep sedation was found to be significantly higher in the IMIE Group compared to the CPIA Group (*P* = 0.018). Among the events interfering with the procedure, the incidence of belching or vomiting was not different between the two groups. However, patients showing spontaneous movement or those requiring physical restraint due to uncooperative behavior were observed more often in the IMIE Group compared to the CPIA Group (*P* = 0.024 and 0.006, respectively). In addition, there was no difference between groups with regard to the incidence of other respiratory events or hemodynamic events. There was no serious adverse event in both groups.

**Table 6:** Adverse events.

**Variable**	**IMIE**	**CPIA**	***P* value**
	**(n = 76)**	**(n = 78)**	
Patients with at least one episode of unintended deep sedation (MOAA/S 0 ~ 2), n (%)	13 (17.1)	4 (5.1)	0.018
Events interfering with procedure, n (%)			
Belching	18 (23.7)	16 (20.5)	0.635
Vomiting	1 (1.3)	1 (1.3)	> 0.999
Spontaneous movement	46 (60.5)	33 (42.3)	0.024
Requiring physical restraint	21 (27.6)	8 (10.3)	0.006
Any of the above	51 (67.1)	41 (52.6)	0.066
Respiratory events, n (%)			
Chin lift/jaw thrust maneuver	11 (14.5)	10 (12.8)	0.765
Increase in O_2_ flow	2 (2.6)	6 (7.7)	0.276
Assisted mask ventilation	1 (1.3)	2 (2.6)	> 0.999
Any of the above	11 (14.5)	11 (14.1)	0.948
Hemodynamic events, n (%)			
Hypertension	15 (19.7)	22 (28.2)	0.219
Hypotension	3 (3.9)	2 (2.6)	0.679
Tachycardia	2 (2.6)	7 (9.0)	0.167
Bradycardia	0 (0.0)	1 (1.3)	> 0.999
Any of the above	19 (25.0)	29 (37.2)	0.103

IMIE, intermittent midazolam/propofol injection by endoscopist; CPIA, continuous propofol infusion by anesthesiologist; MOAA/S, modified observer assessment of alertness/sedation.

## Discussion

Providing sedation during GI endoscopic procedures has now become standard of care [18,21], and despite much controversy, propofol has gained popularity as the sedative of choice by many endoscopists as well as anesthesiologists [9–11,22,23]. As of now, whether or not to use propofol during sedation for GI endoscopy no longer seems to be an issue. Rather, who and how that person should administer propofol in order to maximize the clinical outcomes relevant to sedation method are hotly debated. Compared to the abundance of studies focused on safety outcomes, little is known on the difference in ESD performance according to method of sedation. To the best of our knowledge, the present study is the first to investigate how sedation method during ESD affects the satisfaction levels of the endoscopists.

It is important to acknowledge the components of the two different sedation methods used in this study. The IMIE Group basically employs the method of intermittent bolus administrations of propofol supplemented by bolus doses of meperidine for pain control, which are given by an endoscopist. While propofol is also used as the main sedative, the CPIA Group differs from the IMIE Group in that both propofol and remifentanil is administered by continuous infusion, a method often advocated by anesthesiologists for monitored anesthesia care [24].

Although continuous propofol infusion with opioid administration by an anesthesiologist had been previously suggested as a risk factor for post-ESD pneumonia by Park *et al*. [25], the depth of sedation was not controlled in their study. Another study reported significantly slower recovery times and a higher incidence of hypotension with continuous propofol infusion by a trained nurse, when deep sedation was targeted during GI endoscopy [26]. In contrast, overall quality of sedation, operating conditions and clinical recovery profiles were found to be comparable between intermittent bolus injection, variable-rate continuous infusion and target-controlled infusion of propofol when a moderate depth of sedation was targeted during monitored anesthesia care for breast biopsy [27]. Taken together with the results of the present study, a strictly controlled moderate level of sedation may be of vital importance in achieving better procedural outcome. The proportion of patients that showed at least one episode of unintended deep sedation was significantly greater in the IMIE Group compared to the CPIA Group in this study. While this may be partially attributable to the familiarity with titrating drug infusion rates and the ability to maintain a more stable level of sedation of the anesthesiologist, the difference in opioids used in each group should also be taken into account. Patel *et al*. [28] reported that the incidence of unintended deep sedation during sedation with midazolam and meperidine was as high as 85% during endoscopic retrograde cholangiopancreatography (ERCP). They also found a more frequent occurrence of deep sedation with advanced endoscopic procedures such as ERCP and endoscopic ultrasound, compared to simple diagnostic procedures such as esophagogastroduodenoscopy and colonoscopy. This was assumed to be probably related to greater total sedative-analgesic doses during prolonged complex procedures and the pharmacokinetics of midazolam and meperidine. Midazolam and meperidine both have slower onset times and longer half-lives compared to propofol and remifentanil [6,29], which will render the former combination of drugs more prone to a slower-than-desired speed of titration and the tendency for oversedation.

The speed of recovery was found to be significantly faster in the CPIA Group, with more patients showing full recovery within 5 minutes after ESD. This also seems to be attributable to the aforementioned difference in agents, rather than method of drug delivery. Hayee *et al*. [30] found that recovery time was significantly shorter in patients receiving fentanyl than those receiving meperidine for sedation with midazolam during colonoscopy. Considering that the half-life of remifentanil is even shorter that fentanyl [29], the faster recovery in the CPIA Group compared to the IMIE Group seems natural.

The most interesting aspect of this study seems to be the contradicting results of the satisfaction levels of the endoscopists and the patients. The satisfaction of the endoscopists during procedures that are performed under moderate sedation can be expected to be determined largely by the cooperativeness of the patient and the overall stability of sedation. An uncooperative patient showing sudden movements will hinder the safety and precision of the procedure, and may require physical restraint in order to secure the patient. A cooperative patient can be easily judged to be one under bearable circumstances, if not completely comfortable. In terms of events interfering with the procedure, the incidence of spontaneous movements of the patients, and more importantly, patients requiring physical restraint were significantly lower in the CPIA Group. However, the satisfaction levels of the endoscopists and the patients are opposing in the present study, which seem to contradict the common perception of a ‘cooperative patient’. In addition to decreasing anxiety and discomfort of the patient, amnesia is considered as one of the goals of sedation for endoscopy [31], and this may have affected the satisfaction levels of the patients. Although the patient may seem perfectly comfortable and cooperative during the procedure, the fact that he or she is able to recall the events afterwards may have been a source of dissatisfaction with the whole procedure. A key characteristic of benzodiazepines is their amnestic property [6], and the bolus dose of midazolam that was given in the IMIE Group at the beginning of sedation may have contributed to these results. Adding a small dose of midazolam to the CPIA regimen may possibly help in overcoming this problem.

This study is not without limitations. First of all, the causes of low satisfaction scores in some patients were not evaluated. Although the primary outcome of the present study was the satisfaction levels of the endoscopists, the conflicting results seen in patient satisfaction cannot be ignored. The cause of dissatisfaction of the patients may have been due to several reasons such as lack of analgesia or amnesia, and investigating this aspect would have provided more insight into how the sedation protocols could be improved in the future. Secondly, the sedation providers were different between the two groups. Although one may speculate that a trained anesthesiologist would probably be more skillful in procedural sedation, the difference in sedative regimen and administration technique cannot be ruled out as contributing factors. A randomized trial comparing only the regimen itself or sedation provider is needed to discern the major factor affecting the results. Another important limitation is that most of the endoscopists were aware of the sedation method during ESD. This study had been initially planned to be carried out with both the patients and endoscopists blinded to the sedation method, but this proved to be practically impossible in the clinical setting. Concealing the identity of the clinician providing sedation from the endoscopist was extremely difficult, and thus only the patient remained completely blinded to group allocation. Although this may have been a source of bias in the reporting of satisfaction scores, the endoscopists were informed to be as objective as possible in reporting their opinions. Finally, although this study showed the comparisons of sedation-related outcomes between the two sedation methods during gastric ESD, it is unclear whether the results of the study are applicable to other ESD procedures. For example, colonic ESD requires greater endoscopic skill compared to gastric ESD because of anatomical features of the colon including its longer length, narrow lumen, extensive flexion and thinner walls [32]. More studies on ESD for gastrointestinal lesions other than gastric neoplasms are needed to generalize about our results.

In conclusion, this study shows that continuous propofol and remifentanil infusion by an anesthesiologist during ESD can increase the satisfaction levels of the endoscopists by providing a more stable state of sedation. These results, however, should be cautiously interpreted because the endoscopists were aware of sedation methods during ESD. Together with this finding, a further investigation on improving patient satisfaction may be able to offer supporting evidence for a more ideal sedation regimen for smooth ESD procedures.

## Supporting Information

S1 Case report form Sample case report form. (DOC)

S1 Checklist CONSORT checklist of the study. (DOC)

S1 Informed consent form Sample informed consent form. (DOC)

S1 Protocol Clinical research protocols (English version). (DOCX)

S2 Protocol Clinical research protocols (original language version). (DOCX)
