# Correction: Assessing the Stability and Safety of Procedure during Endoscopic Submucosal Dissection According to Sedation Methods: A Randomized Trial

**DOI:** 10.1371/journal.pone.0127473

**Published:** 2015-04-29

**Authors:** The PLOS ONE Staff

The last author, Young Chul Yoo, should be noted as Corresponding Author for this work. His email address is seaoyster@yuhs.ac. The publisher apologizes for the error.
