# Supplementary material for: Assessing the Stability and Safety of Procedure during Endoscopic Submucosal Dissection According to Sedation Methods: A Randomized Trial
Source: PLoS One. 2015 Mar 24;10(3):e0120529. doi: 10.1371/journal.pone.0120529 (PMC4372558; doi:10.1371/journal.pone.0120529)
Supplement: S1 Case report form — (DOC) [file pone.0120529.s001.doc]

**CASE REPORT FORM**

진정 방법에 따른 내시경 점막하 박리술의 시술 성적 평가

CRF Version: 1.3 (2013/03/27)

스크리닝 번호 [ ]

일련 번호 [ ]

피험자 이니셜 [ ]

**Screening**

**방문일: [ ]년 [ ]월 [ ]일**

**본 환자(또는 법정 대리인)가 동의서에 서명하였습니까?**

**[ ]Yes 🡺 서면 동의일: [ ]년 [ ]월 [ ]일**

**[ ]No 🡺 임상시험에 참여할 수 없습니다.**

**생년월일: [ ]년 [ ]월 [ ]일**

**성별: [ ] 남 [ ]여**

**흡연력**

**[ ] 지금까지 전혀 흡연한 적이 없음 (non-smoker)**

**[ ] 현재 흡연하고 있지 않으며 지금까지 흡연한 담배가 총 100 개피 (5갑) 를 넘지 않음 (non-smoker)**

**[ ] 현재는 흡연하지 않고 있으나 지금까지 흡연한 담배가 총 100 개피 (5갑) 을 넘음 (ex-smoker)**

**[ ] 현재 흡연 중임 (current smoker)**

**과거병력**

**[ ]고혈압 [ ]심장질환 [ ]당뇨**

**[ ]간염 [ ]뇌경색 [ ]암**

**ASA Physicial Status**

**[ ]정상 (ASA physical status 1)**

**[ ]경도의 전신 질환 동반 (ASA physical status 2)**

**[ ]심각한 전신 질환 동반 (ASA physical status 3)**

**[ ]항시 생명을 위협할 만한 전신 질환 동반 (ASA physical status 4)**

**[ ]수술이 아니면 생존하기 어려운 상태 (ASA physical status 5)**

**[ ]뇌사 상태 (ASA physical status 6)**

**항혈소판제 혹은 항응고제 복용**

**[ ]복용 : (복용시 복용 약제명: )**

**[ ]미복용**

**Screening**

**내시경 점막하 박리술을 시행할 병변 개수 [ ] 개**

**병변의 위치**

**[ ]Upper thirid (fundus, cardia, upper body)**

**[ ]Middle third (mid body, lower body)**

**[ ]Lower third (angle, antrum, pylorus)**

**병변의 모양 (내시경 육안 소견)**

**[ ]Elevated**

**[ ]Flat**

**[ ]Depressed**

**병변 크기 (내시경 육안 소견)**

**[ ] mm**

**병변 침윤층 (내시경 육안 소견)**

**[ ]T1a (점막 내 침윤)**

**[ ]T1b (점막하층 침윤)**

**[ ]T2 (고유근층 침윤)**

**병변 궤양 동반 여부**

**[ ]동반**

**[ ]미동반**

**병변 조직검사 결과**

**[ ]Dysplasia**

**[ ]Well-differentiated adenocarcinoma**

**[ ]Moderate-differentiated adenocarcinoma**

**[ ]Poorly-differentiated adenocarcinoma**

**[ ]Signet ring cell carcinoma**

**[ ]기타**

**내시경 점막하 박리술 시술 예정자 [ ]**

**Screening**

**문진 (이상 소견이 있는 경우 기재)**

**신체 검사 (이상 소견이 있는 경우 기재)**

**ECOG status**

**[ ]0 [ ]1 [ ]2 [ ]3 [ ]4**

**신장 [ ] 체중 [ ]**

**혈압 [ ] 맥박 [ ]**

**호흡수 [ ] 체온 [ ]**

**실험실적 검사**

**WBC [ ] Neutrophil(%) [ ]**

**Hemoglobin [ ] Platelet [ ]**

**PT(INR) [ ] aPTT [ ]**

**Protein [ ] Albumin [ ]**

**AST [ ] ALT [ ]**

**Total bilirubin [ ]**

**BUN [ ] Creatinine [ ]**

**Sodium [ ] Potassium [ ]**

**Chloride [ ]**

**흉부 X선 검사 소견 (내시경 점막하 박리술 시행 전)**

**[ ]**

**심전도**

**Heart rate [ ]bpm**

**PR interval [ ]msec QRS duration [ ]msec**

**QT interval [ ]msec QTc [ ]msec**

**검사결과 [ ]**

**Screening**

**선정기준 (다음 항목이 모두 “예” 이면, 본 피험자는 임상 시험에 참여할 수 있다.)**

**1. 만 20 세 이상, 혹은 80 세 미만의 환자 [ ]예 [ ]아니오**

**2. 병변이 다음 중 한 가지 이상 만족 [ ]예 [ ]아니오**

**(1) 크기와 상관없이 궤양을 동반하지 않고 점막 내에 국한된 것으로 판단되는 well- 혹은 moderate-differentiated adenocarcinoma**

**(2) 궤양 동반 여부와 상관없이 점막 내에 국한된 것으로 판단되는 크기 3 cm 이하의 well- 혹은 moderate-differentiated adenocarcinoma**

**(3) 궤양을 동반하지 않고 점막 내에 국한된 것으로 판단되는 크기 2 cm 이하의 adenocarcinoma 혹은 signet ring cell carcinoma**

**(4) 크기 2 cm 이상의 dysplasia**

**3. ECOG performance status 0 혹은 1 [ ]예 [ ]아니오**

**4. ASA physical status 1~3 [ ]예 [ ]아니오**

**5. 본 임상 시험에 참여할 것을 자발적으로 결정하고 임상시험 준수사항을 잘 이행할 것을 서면 동의함 [ ]예 [ ]아니오**

**Screening**

**제외기준 (다음 조건의 어느 하나라도 해당되는 피험자는 임상 시험에 참여할 수 없다.)**

**1. 이전에 부분 위절제술을 시행 받은 적이 있음 [ ]예 [ ]아니오**

**2. 이전에 위루술을 시행 받은 적이 있음 [ ]예 [ ]아니오**

**3. 내시경 점막하 박리술을 시행할 병변이 이전에 내시경적 절제술을 시행했던 병변임 [ ]예 [ ]아니오**

**4. 내시경 점막하 박리술을 시행할 병변이 세 개 이상임**

**[ ]예 [ ]아니오**

**5. 다른 시술로 인해 ESD 전 24시간 이내에 진정을 시행 받을 예정인 자임**

**[ ]예 [ ]아니오**

**6. 임신 혹은 수유 중임 [ ]예 [ ]아니오**

**7. 본 연구에 사용하는 약제에 allergy 과거력이 있음**

**[ ]예 [ ]아니오**

**8. 심각한 신경학적 또는 정신적 동반 질환(간질 또는 치매 등)이 있음**

**[ ]예 [ ]아니오**

**9. 환자 및 보호자의 고지된 동의 (informed consent)를 획득하지 못한 환자**

**[ ]예 [ ]아니오**

**Screening**

**적합성 여부**

**본 환자는 임상 시험에 적합합니까?**

**[ ]예**

**일련번호 기재 [ ]**

**무작위 배정 [ ]IMIE군 [ ]CPIA군**

**[ ]아니오**

**부적합 사유 [ ]선정/제외기준 부적합**

**[ ]동의철회**

**[ ]기타: 상세사유( )**

**시험자 서명**

**본 증례기록서의 모든 항목을 검토하였으며 빠짐없이 정확하게 기재하였음을 확인합니다.**

**서명: 서명일: [ ]년 [ ]월 [ ]일**

**Admission for ESD**

**시술 성공 여부 [ ]예 [ ]아니오**

**진정 시작 시각 [ ]시 [ ]분**

**내시경 점막하 박리술 시작 시각**

**[ ]시 [ ]분**

**MOAAS scale (0 ~ 6점)**

**시술 시작 시 (진정 유도 후 내시경 삽입 전)**

**[ ]시 [ ]분 [ ]점 (최소 1회 이상 체크)**

**[ ]시 [ ]분 [ ]점 (필요시 추가 체크)**

**[ ]시 [ ]분 [ ]점 (필요시 추가 체크)**

**[ ]시 [ ]분 [ ]점 (필요시 추가 체크)**

**시술 중 (내시경 삽입 후, incision 전)**

**[ ]시 [ ]분 [ ]점 (최소 1회 이상 체크)**

**[ ]시 [ ]분 [ ]점 (필요시 추가 체크)**

**[ ]시 [ ]분 [ ]점 (필요시 추가 체크)**

**[ ]시 [ ]분 [ ]점 (필요시 추가 체크)**

**시술 중 (incision 후, dissection 전)**

**[ ]시 [ ]분 [ ]점 (최소 1회 이상 체크)**

**[ ]시 [ ]분 [ ]점 (필요시 추가 체크)**

**[ ]시 [ ]분 [ ]점 (필요시 추가 체크)**

**[ ]시 [ ]분 [ ]점 (필요시 추가 체크)**

**시술 종료 시 (dissection 후 진정 종료 전)**

**[ ]시 [ ]분 [ ]점 (최소 1회 이상 체크)**

**[ ]시 [ ]분 [ ]점 (필요시 추가 체크)**

**[ ]시 [ ]분 [ ]점 (필요시 추가 체크)**

**[ ]시 [ ]분 [ ]점 (필요시 추가 체크)**

**활력 징후 (5분 간격 측정) [ SBP / DBP, PR , RR, SpO2(%) ]**

**[ ]시 [ ]분 [ ]**

**[ ]시 [ ]분 [ ]**

**[ ]시 [ ]분 [ ]**

**[ ]시 [ ]분 [ ]**

**[ ]시 [ ]분 [ ]**

**[ ]시 [ ]분 [ ]**

**[ ]시 [ ]분 [ ]**

**[ ]시 [ ]분 [ ]**

**[ ]시 [ ]분 [ ]**

**[ ]시 [ ]분 [ ]**

**[ ]시 [ ]분 [ ]**

**[ ]시 [ ]분 [ ]**

**[ ]시 [ ]분 [ ]**

**[ ]시 [ ]분 [ ]**

**[ ]시 [ ]분 [ ]**

**[ ]시 [ ]분 [ ]**

**[ ]시 [ ]분 [ ]**

**[ ]시 [ ]분 [ ]**

**[ ]시 [ ]분 [ ]**

**[ ]시 [ ]분 [ ]**

**[ ]시 [ ]분 [ ]**

**[ ]시 [ ]분 [ ]**

**[ ]시 [ ]분 [ ]**

**[ ]시 [ ]분 [ ]**

**[ ]시 [ ]분 [ ]**

**[ ]시 [ ]분 [ ]**

**[ ]시 [ ]분 [ ]**

**[ ]시 [ ]분 [ ]**

**[ ]시 [ ]분 [ ]**

**[ ]시 [ ]분 [ ]**

**[ ]시 [ ]분 [ ]**

**[ ]시 [ ]분 [ ]**

**[ ]시 [ ]분 [ ]**

**[ ]시 [ ]분 [ ]**

**시술이 방해되는 사건 (총 횟수 측정)**

**Belching [ ]**

**Vomiting [ ]**

**Spontaneous moving [ ]**

**Physical restraint [ ]**

**진정 수준이 높아지는 것과 연관되는 사건 (총 횟수 측정)**

**Increased O_2_ flow [ ]**

**Chin lift [ ]**

**O_2_ mask apply [ ]**

**Ambu-bagging [ ]**

**Intubation [ ]**

**시술 중 출혈 여부**

**[ ]예 [ ]아니오**

**내시경 점막하 박리술 종료 시각 [ ]시 [ ]분**

**진정 종료 시각 [ ]시 [ ]분**

**시술 종료 후 경과된 시간에 따른 회복 여부**

**시술 종료 5분 후 회복 [ ]예 [ ]아니오**

**시술 종료 10분 후 회복 [ ]예 [ ]아니오**

**실제 진정 수준**

**[ ]Minimal sedation**

**[ ]Moderate sedation**

**[ ]Deep sedation**

**[ ]General anesthesia**

**진정에 사용한 약제 용량**

**Midazolam (Midazolam®) [ ]mg**

**Propofol (Pofol®) [ ]mg**

**Fentanyl (Fentanyl®) [ ]μg**

**Remifentanil (Ultiva®) [ ]μg**

**내시경 점막하 박리술 후 시술자의 만족도 (시술 종료 직후에 체크)**

**시술 시 사용한 진정 방법에 대한 만족도 (0 ~ 10점) [ ]점**

**내시경 점막하 박리술 후 환자의 통증 (진정에서 회복된 직후 회복실에서 체크)**

**Visual analogue scale (VAS) (0 ~ 10점) [ ]점**

**내시경 점막하 박리술 시술 당일 흉부 X선 검사**

**흉부 X선 검사 소견 [ ]**

**Admission for ESD**

**내시경 점막하 박리술 후 통증 및 만족도 (시술 후 1일째 아침에 체크)**

**Visual analogue scale (VAS) (0 ~ 10점) [ ]점**

**시술에 대한 전반적인 만족도 (0 ~ 10점) [ ]점**

**시술 시 사용한 진정 방법에 대한 만족도 (0 ~ 10점) [ ]점**

**내시경 점막하 박리술 시술 후 1일 째 혈액학적 검사 및 흉부 X선 검사**

**WBC [ ] Neutrophil(%) [ ]**

**Hemoglobin [ ] Platelet [ ]**

**흉부 X선 검사 소견 [ ]**

**내시경 점막하 박리술 후 병리 결과**

**조직학형**

**[ ]dysplasia**

**[ ]well-differentiated adenocarcinoma**

**[ ]moderate-differentiated adenocarcinoma**

**[ ]poorly-differentiated adenocarcinoma**

**[ ]signet ring cell carcinoma**

**[ ]기타**

**침윤 깊이**

**[ ]pT1a**

**[ ]pT1b, 점막근판 500 μm 이하의 점막하층 침윤**

**[ ]pT1b, 점막근판 500 μm 이상의 점막하층 침윤**

**[ ]pT2**

**[ ]pT3**

**절제면의 암 세포 침윤**

**Horizontal margin [ ]Positive [ ]Negative**

**Vertical margin [ ]Positive [ ]Negative**

**절제면의 이형성 세포 침윤**

**Horizontal margin [ ]Positive [ ]Negative**

**Vertical margin [ ]Positive [ ]Negative**

**림프 혈관계 침범 [ ]있음 [ ]없음**

**병변 크기 (병리검사결과로 보고된 병변의 크기)**

**장축 [ ] mm 단축 [ ] mm**

**내시경 점막하 박리술 시술 결과**

**일괄절제 여부 [ ]예 [ ]아니오**

**완전절제 여부 [ ]예 [ ]아니오**

**치료적절제 여부 [ ]예 [ ]아니오**

**내시경 점막하 박리술 합병증**

**시술후 출혈 [ ]예 [ ]아니오**

**천공 [ ]예 [ ]아니오**

**흡인성 폐렴 [ ]예 [ ]아니오**
