# Supplementary material for: Assessing the Stability and Safety of Procedure during Endoscopic Submucosal Dissection According to Sedation Methods: A Randomized Trial
Source: PLoS One. 2015 Mar 24;10(3):e0120529. doi: 10.1371/journal.pone.0120529 (PMC4372558; doi:10.1371/journal.pone.0120529)
Supplement: S1 Informed consent form — (DOC) [file pone.0120529.s003.doc]

**피험자를 위한 설명서**

**임상시험의 명칭:** 진정 방법에 따른 내시경 점막하 박리술의 시술 성적 평가

**서론**

귀하는 위암 혹은 위선종에 대해 내시경 점막하 박리술을 시행할 때 진정 방법에 따른 시술 성적을 평가하는 임상시험에 참여하시도록 제안을 받았을 것입니다. 이 설명문은 귀하가 이 시험에 참여할 것인지를 결정하는 데 도움이 되도록 정보를 제공하기 위한 것으로서 본 임상시험에 대한 전반적인 정보를 포함하고 있습니다. 만일 본 내용 이외에도 궁금한 것이 있거나 혹은 본 내용에 대해 의문이 있을 때에는 담당의사에게 질문하고 논의할 수 있는 기회를 갖게 될 것입니다. 본 임상시험에 대한 참여 여부는 귀하가 자발적으로 결정하는 것이며, 만일 참여에 동의하신다면 첨부된 피험자 동의서에 서명하시면 됩니다. 서명한 후에는 본 동의설명서와 서명한 동의서 사본을 1 부씩 가지게 될 것입니다.

**이 임상시험의 목적은 무엇입니까?**

위암 혹은 위선종에 대해 최소 침습적인 치료를 하기 위해 점차 내시경적 절제술의 사용 빈도가 늘고 있는 추세입니다. 내시경 점막하 박리술과 같은 내시경적 절제술을 시행하기 위해서는 시술 중 환자의 진정이 매우 중요한데, 아직까지 어떠한 진정 방법이 더 나은가에 대해서는 충분한 연구가 이뤄진 바가 없고, 따라서 진정 방법의 표준은 정해져 있지 않은 것이 현실입니다. 본 연구에서는 위암 혹은 위선종에 대해 내시경 점막하 박리술을 시행할 때 진정 방법에 따라 시술 성적이 차이가 나는지를 확인하여 적절한 진정 방법을 제시하고자 함입니다.

**진정 방법에 사용하는 약제는 어떤 것입니까?**

진정 방법은 내시경 의사에 의해 진정을 시행하는 경우와 마취과 의사에 의해 진정을 시행하는 경우로 나뉘어집니다. 내시경 의사에 의해 진정을 할 때에는 미다졸람이라는 진정제를 기본으로 하여 통증 완화를 위해 페치딘이라는 진통제를 사용하며 필요에 따라 속효성 진정제인 프로포폴을 추가로 사용하게 됩니다. 마취과 의사에 의해 진정을 할 때에는 프로포폴을 기본으로 하여 통증 완화를 위해 레미펜타닐이라는 모르핀 계열 진통제를 사용하게 됩니다. 이들 약제는 모두 내시경 시술 시 안전하게 사용할 수 있는 진정 혹은 진통제임이 널리 알려져 있습니다. 물론, 저산소증이나 호흡 부전과 같이 생명을 위협할 수 있는 부작용도 드물게 나타날 수는 있습니다만, 진정제를 사용하지 않고 내시경 점막하 박리술을 시행하는 것은 불가능에 가까워 진정제 사용이 불가피하며, 진정 중 설령 부작용이 나타난다 하더라도 연구진들은 이러한 부작용 가능성을 항시 염두에 두고 있기 때문에 적절한 응급 처치를 시행할 수 있습니다. 현재 본원에서는 마취과 의사에 의해 진정을 시행하는 것을 표준으로 하고 있으나 본원과 유사한 규모의 타 기관에서는 내시경 의사에 의해 진정을 시행하는 것을 표준으로 하고 있습니다. 하지만, 앞 항목에서 언급하였다시피 내시경 점막하 박리술을 위한 진정 방법의 국제적 표준은 없는 실정으로 귀하가 본 임상 시험에 참여하게 되면 두 가지 방법 중 하나로 진정을 시행 받게 될 것입니다.

**본 임상시험에 참여하는 환자수와 참여기간은 얼마나 됩니까?**

본 임상시험에는 약 157명의 환자가 모집될 것입니다. 임상시험에 참여하는 피험자는 그렇지 않은 환자와 마찬가지로 내시경 점막하 박리술 후 동일한 스케줄로 추가 검사 (혈액검사, X-ray)를 시행 받고 퇴원하게 되며 보통 3박 4일 전후의 입원 기간 소요됩니다. 이후 1개월 뒤까지 외래에서 합병증 발생 여부를 확인하게 되며 귀하가 참여하는 임상시험은 이 때 종료하게 됩니다. 본 연구는 세브란스병원 연구심의위원회 승인 후 최대 24개월까지 진행될 예정입니다.

**임상시험과정**

◆ 본 임상시험에 적격한 피험자임을 결정하기 위한 선별방문

귀하가 본 연구에 참여할 것을 동의하면, 혈압측정, 혈액검사, 흉부 X-ray, 심전도 검사, 그리고 과거의 병력, 신체상태, 병용약물 등을 확인하여 본 연구에서 정하는 조건에 맞는지 알아보는 과정을 겪을 것입니다. 귀하가 본 연구 참여에 적합하다면 연구 참여를 결정하게 됩니다. 연구 참여가 결정되면 시험에 참여하는 157 명 중의 한 명이 되며, 내시경 점막하 박리술 시 사용할 진정 방법을 무작위 배정을 통해 정하게 됩니다. 무작위 배정은 컴퓨터로 추출된 난수표에 의해 정해지며, 확률은 정해져 있지만 앞 면이 나올 지 뒷 면이 나올지 알 수 없는 동전 던지기와 마찬가지로 귀하가 어느 진정 방법을 시행 받을 지 미리 알 수는 없습니다.

◆ 내시경 점막하 박리술 시행

귀하가 연구에 참여하게 됨으로써 내시경 점막하 박리술 시 사용할 진정 방법은 무작위로 배정 받게 되지만, 이 외에 내시경 점막하 박리술 전, 후 시행하는 혈액검사, 흉부 X-ray, 심전도 검사 등과 내시경 점막하 박리술 시술 과정 등은 연구에 참여하지 않을 때와 모두 동일합니다. 내시경 점막하 박리술은 병변의 위치, 크기, 깊이 등 난이도에 따라 짧게는 30분, 길게는 수 시간 까지 소요됩니다.

**피험자 준수사항**

귀하의 안전과 정확하고 과학적인 연구에 기여하기 위해서 의료진의 지시를 따라 주시기 바랍니다. 임상시험 기간 중에 귀하가 느끼는 모든 증상에 대해서는 반드시 의사 및 간호사에게 알려야 합니다.

**임상시험 참여자의 권리**

귀하가 임상시험에 참여하는 것은 자유의사에 의한 것이며 중단하고 싶을 때는 언제든지 이유를 말하지 않고도 임상시험 참여를 거부할 수 있으며 임상시험을 중단할 수 있습니다. 귀하가 임상시험 참여를 철회하더라도 귀하가 다른 의학적 치료를 받는 데는 아무런 영향이 없습니다. 임상시험담당자(의사)는 시험기간 중에 발견된 모든 결과를 당신에게 알려줄 것이며 이 결과가 시험을 계속 참여해야 할 지 당신의 의지에 영향을 줄 수도 있을 것입니다.

**임상시험 도중 참여가 중지되는 경우 및 그 사유는 무엇입니까?**

귀하가 정확한 병력을 제공하지 않았거나 임상시험의 지침이나 임상시험실시기관의 규정을 따르지 않은 것으로 밝혀지면 귀하는 언제라도 임상시험에서 탈락될 수 있습니다. 그 외에 임상시험에 등록되었지만, 어떠한 이유에서든 임상시험 전체 기간에 참여할 수 없는 경우는 본 시험에서 탈락됩니다. 그 자세한 경우는 다음과 같습니다.

환자 또는 환자의 보호자가 임상시험의 중단을 요구하는 경우

중증의 이상반응으로 인하여 시험 지속이 곤란한 경우

중증의 합병증 발현으로 시험 지속이 곤란한 경우

시험진행 도중 선정기준 및 제외기준 위반이 발견된 경우

병용금지약제를 사용한 경우

환자 추적이 불가능한 경우

기타 담당의사가 판단했을 때 시험 지속이 곤란하다고 판단한 경우

**본 시험에 참여함으로써 얻게 되는 이익은 무엇입니까?**

본 시험에 사용하는 두 가지 진정 방법은 모두 기존에 내시경 시술 시 널리 사용하는 진정 방법이며 시술과 연관되어 시행되는 혈액 검사, 흉부X-ray 및 심전도 검사 등은 모두 정상적인 시술 과정에서 필요한 것들로서 이들 검사 및 처치에 대한 비용은 모두 피험자 본인이 부담하게 됩니다. 또한, 마취과 의사에 의해 진정을 시행하는 경우에는 그에 따른 시술 비용이 추가되므로 진정 방법에 따라 비용 차이가 발생할 수 있습니다.

또한, 어느 진정 방법이 배정된다 하더라도 다른 방법에 비해 내시경 점막하 박리술의 시술 성적이 나을지에 대해 보장할 수는 없습니다. 하지만, 이 임상시험은 추후 내시경 점막하 박리술을 시행 받는 귀하와 비슷한 다른 환자들에게 효과적인 진정 방법을 제시하는데 도움을 줄 수 있을 것입니다.

**본 시험에 참여함으로써 얻게 되는 금전적 보상은 무엇입니까?**

귀하가 시험에 참여함으로써 받을 수 있는 금전적 보상은 없습니다.

**본 시험에 참여 중 피해 발생 시 보상 대책이 있습니까?**

내시경 점막하 박리술은 기본적으로 합병증을 수반할 수 있는 의료 시술이며 본 시험 참여 중에도 호흡 부전, 출혈, 천공, 폐렴 등 합병증 발생 가능성은 있지만, 본 시험에서 이뤄지는 모든 시술 절차는 귀하가 임상시험에 참여하지 않게 되더라도 동일하게 진행되는 과정으로 피해 발생 시 보상은 따로 없습니다. 물론, 시험 도중 발생하는 직, 간접적 상해에 대해서는 귀하의 담당 의사가 상해의 치료를 위해 최선의 조치를 취할 것입니다.

**신분의 비밀보장에 관하여는 다음과 같습니다.**

귀하의 신상 및 본 임상시험의 모든 결과는 병원에 기록, 보관될 것이며 진정 방법에 관한 유효성을 평가하기 위한 목적으로만 검토될 것이고, 모든 자료는 엄격히 비밀이 유지되며 보호를 받게 됩니다. 귀하가 본 임상시험에 참가하기로 동의할 경우 본 시험을 통해 귀하에 관해 수집된 자료는 익명으로 다루어집니다. 시험과 관련하여 의문사항이 있을 경우 언제든지 담당의사에게 문의할 수 있습니다.

귀하는 본 동의서에 서명함으로써 모니터요원, 점검을 실시하는 자, 심사위원회 및 식품의약품안전청장의 관련 규정이 정하는 범위에서 임상시험의 실시 절차와 자료의 신뢰성을 검증하기 위해 본인의 의무기록을 직접 열람하는 데에 동의하신 것입니다. 물론 이 자료들은 위에 기재된 사람들이 임상시험에 관계된 그들의 의무사항을 수행하는 것과 관련해서만 사용될 것입니다

또한 귀하의 건강에 중요하거나 이 임상시험에 계속 참여하겠다는 귀하의 의사에 영향을 미칠 수 있는 모든 새로운 정보를 귀하 혹은 귀하의 법정대리인에게 알려줄 것입니다.

**응급시의 연락처**

본 연구에 관하여 궁금한 점이 있거나 연구와 관련이 있는 상해가 발생한 경우에는 아래의 연구자에게 연락하여 주십시오.

연구자 성명 : 박 준 철

연구자 주소 : 서울시 서대문구 연세로 50 세브란스병원 소화기내과

임상시험에 참여하는 피험자로서 윤리적인 문제나 귀하의 권리에 대하여 질문이 있는 경우에는 연구자에게 말씀하시거나 다음의 번호로 문의하실 수 있습니다.

세브란스병원 연구심의위원회 : 02-2228-0430~4

세브란스병원 임상연구보호센터 : 02-2228-0450~4

**피험자 동의서**

**임상시험의 명칭:** 진정 방법에 따른 내시경 점막하 박리술의 시술 성적 평가

**병원명 / 연구책임자: 세브란스 병원 / 박 준 철**

**피험자 이니셜 / 스크리닝 번호:** _______________________________________

본인은 내시경 점막하 박리술에 사용되는 진정 방법에 관한 연구인 본 임상시험의 성격, 수행과정, 이점, 위험성에 대하여 시험자 ______________로부터 설명 받았음을 확인합니다. 또한 본 임상시험이 연구목적으로 수행된다는 사실을 확인하였으며, 임상시험에 관한 서면정보(피험자를 위한 설명서)를 받아 읽어보았고 이해하였으며 본인 혹은 대리인이 서명한 동의서의 사본을 제공받았습니다.

본인은 성별, 나이, 생년월일, 이름의 이니셜, 진단명에 관한 개인적인 세부사항을 포함한 임상시험의 결과가 임상시험 결과보고서에 익명으로 기재될 것임을 알고 있습니다.

본인은 어떤 단계에서도 기득권을 침해 받지 않고 이번 임상시험의 참여와 동의를 철회할 수 있습니다.

본인은 질문할 기회가 충분히 있었으며, 이번 임상시험에 참여할 준비가 되어 있음을 본인의 자유의지로 알립니다.

피험자 성명 : (서명 / 인) 년 월 일

법정 대리인 성명 : (서명 / 인) 년 월 일

(필요한 경우) 피험자와의 관계 :

입회자(증인) 성명 : (서명 / 인) 년 월 일

연구책임자 성명 : (서명 / 인) 년 월 일

(담당자)

* 대리인 : 피험자의 친권자, 배우자, 후견인, 피험자가 임상시험 참여여부를 결정할 수 없는 경우

** 입회자 : 피험자 혹은 대리인이 동의서 서식, 피험자 설명서 및 기타 문서화된 정보를 읽을 수 없는 경우
