# Supplementary material for: Assessing the Stability and Safety of Procedure during Endoscopic Submucosal Dissection According to Sedation Methods: A Randomized Trial
Source: PLoS One. 2015 Mar 24;10(3):e0120529. doi: 10.1371/journal.pone.0120529 (PMC4372558; doi:10.1371/journal.pone.0120529)
Supplement: S1 Protocol — (DOCX) [file pone.0120529.s004.docx]

**Clinical Research Protocols**

1. Title

Assessment of performance of endoscopic submucosal dissection according to the sedation methods

1. Background

Endoscopic submucosal dissection (ESD) is an advanced technique that enables en bloc resection of superficial tumors in the gastrointestinal tract. When compared to endoscopic mucosal resection (EMR), ESD has markedly improved the rate of en bloc resection, even of large lesions that were previously difficult to treat by EMR.[^1^](#_ENREF_1)^,^[^2^](#_ENREF_2) Although great advances have been made in therapeutic endoscopy, ESD still remains a time consuming procedure and requires great endoscopic skill.[^2^](#_ENREF_2)^,^[^3^](#_ENREF_3) Thus, higher levels of sedation or general anesthesia are required for successful completion as compared with routine endoscopy and other therapeutic endoscopy.[^4^](#_ENREF_4) Although standard sedation methods during ESD are not yet established, most cases of ESD treatment are performed under sedation with midazolam or propofol.[^3-7^](#_ENREF_3) Currently, sedation with propofol is gaining a foot-hold because propofol has proven to be a safe and superior to the benzodiazepine in various endoscopic procedures.[^8-14^](#_ENREF_8) Additionally, two prospective studies have demonstrated that sedation with continuous infusion propofol is safe compared to intermittent midazolam injection.[^4^](#_ENREF_4)^,^[^15^](#_ENREF_15) However, the question of whether propofol is administered by endoscopist or anesthesiologist is a highly controversial topic. The product label, approved by the U.S. Food and Drug Administration, states, “Propofol should be administered only by persons trained in the administration of general anesthesia.”[^16^](#_ENREF_16) The American Gastroenterological Association, however, supports that, if undertaken appropriately, gastroenterologist-directed propofol sedation is medicolegally reasonable.[^17^](#_ENREF_17) However, the most research for the issue of who should be providing sedation during the endoscopy focused on legal, safety, and enconomic aspects. No study has yet investigated the effects of sedation methods on ESD performance. We aimed to evaluate the relationship between sedation methods and ESD performance, prospectively.

3. Aim

To evaluate the relationship between the ESD performance and the sedation methods

4. Institution and study period

Institution: Yonsei University College of Medicine, Severance Hospital

Study period: Twenty four months from the approval of the IRB

5. Inclusion and exclusion criteria

A. Inclusion criteria

1) Age 20 ~ 80

2) Gastric adenoma or early gastric cancer (EGC) which fulfilled the ESD indication

A. EGC

a) Differentiated (well- to moderate-differentiated) mucosal (T1a) cancer without ulcer regardless of size

b) Differentiated mucosal cancer smaller than 3 cm in diameter regardless of presence of ulcer

c) Mucosal cancer smaller than 2 cm in diameter without ulcer regardless of presence of ulcer

B. Gastric adenoma

가) Gastric adenoma larger than 2 cm in diameter

3) ECOG performance status 0 – 1

4) American Society of Anesthesiologist (ASA) Physical Status 1 – 3 (Table 1)

5) Appropriate patient compliance

6) Patient who has an opportunity to be an informed participant in his/her health care decisions

B. Exclusion criteria

1) Patients who had previously undergone subtotal gastrectomy

2) Patients who had previously undergone gastrostomy

3) Patients receiving repeated ESD

4) Patients presenting with three or more synchronous lesions

5) Patients that had received sedation for another procedure within 24 hours prior to ESD

6) Pregnant or breastfeeding patients

7) Patients with known allergies to eggs, soy beans or sulfites

8) Patients with debilitating neurologic or psychotic disorders

9) Patients with unable to provide informed consent

C. Screening

1) Esophagogastroduodenoscopy and biopsy

2) Systemic organ function

A) Bone marrow: Neutrophil ≥ 1,500 /mm3, Hemoglobin ≥ 9.0 g/dL, Platelet ≥ 100,000 /mm3

B) Coagulability: PT < 1.5 INR, aPTT ≤ 1.5 x [upper limit of the reference rage]

C) Hepatic function: Total bilirubin < 2.0 mg/dL, AST/ALT < 2.5 x [upper limit of the reference range]

D) Renal function: Creatinine ≤ [upper limit of reference range] or CCr ≥ 60 mL/min (by Cockcroft-Gault equation)

E) Electrolyte: normal range of sodium, potassium, and chloride level

D. Withdrawal

Patients who wish to withdraw from the study can withdraw at any time. When the patients wish to withdraw, investigators may contact them by telephone or visiting for asking a reason for withdrawl. If the reasons for withdrawl are adverse events or abnormal laboratory tests, they should be recorded in the clinical research form.

6. Sample size calculation

1. Sample size: 157
2. Calculation

*H*_0_ : Endoscopists’ satisfaction in the intermittent midazolam/propofol injection controlled by endoscopist (IMIE) group = Endoscopists’ satisfaction in the continuous propofol infusion with opioid administration controlled by anesthesiologist (CPIA) group

*H*_1_ : Endoscopists’ satisfaction in the IMIE group ≠ Endoscopists’ satisfaction in the CPIA group

Sample size calculation was performed based on the results of our pilot study.

The results of the pilot study

The IMIE group: 6 satisfaction, 3 average, 1 dissatisfaction

The CPIA group: 8 satisfaction, 1 average, 1 dissatisfaction

Estimated effect size: 0.25 (using a χ2 test with 2 degree of freedom)

Significance level: 0.05, Power: 80%

Drop-out rate: 1%

Calculated sample size: 157

7. Study design & Methods

A. Methods

1) Single center, open, randomized, comparative study for patients who will undergo ESD for gastric adenoma or EGC

2) Patients who provide informed consent were only included after screening according to the inclusion and exclusion criteria

3) Allocation

A) Stratified randomization by histology (gastric adenoma vs. EGC)

B) Blocked randomization

4) Sedation protocol

A) IMIE group

a) Sedation by endoscopists using following drugs: meperidine (pethidine®, Jeil Pharmaceutical Co. Lt., Daegu, Korea), midazolam (midazolam®, Bukwang Pharm. Co. Ltd., Seoul, Korea), and propofol(pofol®, Dongkook Pharm. Co. Ltd., Seoul, Korea)

b) Initial bolus doses of 50 mg of meperidine IM

c) Bolus doses of 0.05 mg/kg of midazolam IV

d) When patients are found to be undersedated with an Modified Observer Assessment of Alertness/Sedation (MOAA/S) scale (Table 2) of 5 or 6, bolus doses of 0.25 mg/kg of propofol IV will be given.

e) When patients showing signs of discomfort or pain such as spontaneous movements while presenting with an MOAA/S score of 3 or 4, additional bolus doses of meperidine 12.5 mg IV will be given.

B) CPIA group

a) Sedation by anesthesiolgists using following drugs: remifentanil (ulitiva®, GlaxoSmithKline, Co. Ltd., Genval, Belgium) and propofol

b) Initial bolus doses of 0.5 μg/kg of remifentanil IV and continuous infusion of remifentanil at 0.08 μg/kg/min

c) Initial bolus doses of 0.5 mg/kg of propofol IV and continuous infusion of propofol at 2 mg/kg/hr

d) When patients are found to be undersedated with an MOAA/S score of 5 or 6, additional bolus doses of 0.25 mg/kg of propofol will be given and the infusion rate will be increased by 0.5 mg/kg/hr.

e) When patients showing signs of discomfort or pain such as spontaneous movements while presenting with an MOAA/S score of 3 or 4, the infusion rate of remifentanil will be increased by 0.02 μg/kg/min.

f) When the mean blood pressure of the patient falls below 60 mmHg or decrease by more than 20% from baseline, or the patient shows desaturation of SpO_2_ <90%, propofol infusion rates will be decreased by 0.5 mg/kg/hr.

5) Sedation depth, vital signs, and other variables

A) Targetted level of sedation: MOAA/S 3 or 4

B) Assessing of the MOAA/S score

1) Just before the insertion of the endoscope

2) After insertion of the endoscope and before the first incision

3) Immediately after the first incision, before the end of dissection

4) At the end of dissection

5) When patients show signs of undersedation or reaction to discomfort and/or pain

C) Check vital signs every 5 minutes

D) Events interfering with procedure

1) Belching

2) Vomiting

3) Spontaneous moving

4) Physical restraint

E) Respiratory events

1) Chin lift

2) Increased O_2_ flow

3) Assisted mask ventilation

4) Intubation

8. Variables

A. Patient related variables

1) Age

2) Sex

3) Smoking history

4) Co-morbidity

5) ASA Class

6) Anti-platelet agents, anticoagulants

다. Tumor-related variables

1) Histology (cancer or adenoma)

2) Macroscopic appearance (elevated, flat, depressed)

3) Tumor location

A) Upper third (fundus, cardia, upper body)

B) Middle third (mid body, lower body)

C) Lower third (angle, antrum, pylorus)

4) Tumor size

5) Ulceration

라. Procedure-related variables

1) En bloc resection

2) Complete resection

3) Curative resection

4) Procedure time

5) Complication

6) Sedation depth

9. End points

가. Primary endpoint: Endoscopists’ satisfaction according to the sedation methods

나. Secondary endpoint

1) ESD performance according to the sedation methods

2) Patients’ satisfaction according to the sedation methods

다. Statistical analysis

1) Analyzing by intention-to-treat method

2) The primary endpoint is assessed by Chi-square test or Fisher’s exact test.

3) The secondary endpoints are assessed by Chi-square test or Fisher’s exact test.

4) No interim analysis

5) When the missing data are present, complete case analysis will be performed.

10. Ethics and regulation

A. This study protocol conformed to the ethical guidelines of the 1975 Helsinki Declaration and International Conference on Harmonisation of Technical Requirements of Pharmaceuticals for Human Use (ICH) Note for Guidance on Good Clinical Practice (ICH, Topic E6, 1995)

B. This study was approved by the Institutional Review Board of Severance Hospital.

C. Compensation

Both of the two sedation methods in the study are currently used in routine clinical practice. There is no financial compensation for patients who participate in this study.

11. References

1. Miyamoto S, Muto M, Hamamoto Y, et al. A new technique for endoscopic mucosal resection with an insulated-tip electrosurgical knife improves the completeness of resection of intramucosal gastric neoplasms. Gastrointest Endosc 2002;55:576-81.

2. Gotoda T, Yamamoto H, Soetikno RM. Endoscopic submucosal dissection of early gastric cancer. J Gastroenterol 2006;41:929-42.

3. Akasaka T, Nishida T, Tsutsui S, et al. Short-term outcomes of endoscopic submucosal dissection (ESD) for early gastric neoplasm: multicenter survey by osaka university ESD study group. Dig Endosc 2011;23:73-7.

4. Yamagata T, Hirasawa D, Fujita N, et al. Efficacy of propofol sedation for endoscopic submucosal dissection (ESD): assessment with prospective data collection. Internal medicine 2011;50:1455-60.

5. Imagawa A, Fujiki S, Kawahara Y, et al. Satisfaction with bispectral index monitoring of propofol-mediated sedation during endoscopic submucosal dissection: a prospective, randomized study. Endoscopy 2008;40:905-9.

6. Lee H, Yun WK, Min BH, et al. A feasibility study on the expanded indication for endoscopic submucosal dissection of early gastric cancer. Surg Endosc 2011;25:1985-93.

7. Ahn JY, Jung HY, Choi KD, et al. Endoscopic and oncologic outcomes after endoscopic resection for early gastric cancer: 1370 cases of absolute and extended indications. Gastrointest Endosc 2011;74:485-93.

8. Heuss LT, Froehlich F, Beglinger C. Changing patterns of sedation and monitoring practice during endoscopy: results of a nationwide survey in Switzerland. Endoscopy 2005;37:161-6.

9. Cohen LB, Wecsler JS, Gaetano JN, et al. Endoscopic sedation in the United States: results from a nationwide survey. The American journal of gastroenterology 2006;101:967-74.

10. Benson A, Cohen LB, Waye JD, et al. Endoscopic sedation in developing and developed countries. Gut and liver 2008;2:105-12.

11. Horiuchi A, Nakayama Y, Hidaka N, et al. Low-dose propofol sedation for diagnostic esophagogastroduodenoscopy: results in 10,662 adults. The American journal of gastroenterology 2009;104:1650-5.

12. Cote GA, Hovis RM, Ansstas MA, et al. Incidence of sedation-related complications with propofol use during advanced endoscopic procedures. Clin Gastroenterol Hepatol 2010;8:137-42.

13. Riphaus A, Rabofski M, Wehrmann T. Endoscopic sedation and monitoring practice in Germany: results from the first nationwide survey. Zeitschrift für Gastroenterologie 2010;48:392-7.

14. Bo L, Bai Y, Bian J, et al. Propofol vs traditional sedative agents for endoscopic retrograde cholangiopancreatography: a meta-analysis. World Journal of Gastroenterology 2011;17:3538-43.

15. Kiriyama S, Gotoda T, Sano H, et al. Safe and effective sedation in endoscopic submucosal dissection for early gastric cancer: a randomized comparison between propofol continuous infusion and intermittent midazolam injection. Journal of gastroenterology 2010;45:831-7.

16. Aisenberg J, Cohen LB, Piorkowski JD. Propofol use under the direction of trained gastroenterologists: an analysis of the medicolegal implications. The American journal of gastroenterology 2007;102:707-13.

17. Matsui N, Akahoshi K, Nakamura K, et al. Endoscopic submucosal dissection for removal of superficial gastrointestinal neoplasms: A technical review. World J Gastrointest Endosc 2012;4:123-36.

18. Cohen LB, Delegge MH, Aisenberg J, et al. AGA Institute review of endoscopic sedation. Gastroenterology 2007;133:675-701.

Table 1. American Society of Anesthesiologist Physical Status Classification System

| ASA Physical Status 1 | A normal healthy patient |
| --- | --- |
| ASA Physical Status 2 | A patient with mild systemic disease  Ex) Well controlled hypertension or DM, pregnancy, old tuberculosis, mild obesity (BMI>25), smoker (without COPD) |
| ASA Physical Status 3 | A patient with severe systemic disease  Ex) 2 or more ASA physical status 2, poorly controlled hypertension or DM, arrhythmia, well-controlled congestive heart failure, stable angina, old heart attack, obesity (BMI>35), chronic renal failure, COPD, cerebral vascular attack, active tuberculosis |
| ASA Physical Status 4 | A patient with severe systemic disease that is a constant threat to life  Ex) Unstable angina, symptomatic COPD, symptomatic congestive heart failure, hepatorenal failure, ESRD, stable sepsis |
| ASA Physical Status 5 | A moribund patient who is not expected to survive without the operation  Ex) multi-organ failure, unstable sepsis, hypothermia, severe coagulopathy |
| ASA Physical Status 6 | A declared brain-dead patient whose organs are being removed for donor purposes |

Table 2. Modified Observer Assessment of Alertness/Sedation Scale[^18^](#_ENREF_18)

| Responsiveness | Score |
| --- | --- |
| Agitated | 6 |
| Responds readily to name spoken in normal tone (alert) | 5 |
| Lethargic response to name spoken in normal tone | 4 |
| Responds only after name is called loudly and/or repeatedly | 3 |
| Responds only after mild prodding or shaking | 2 |
| Does not respond to mild prodding or shaking | 1 |
| Does not respond to deep stimulus | 0 |
