# Supplementary material for: Assessing the Stability and Safety of Procedure during Endoscopic Submucosal Dissection According to Sedation Methods: A Randomized Trial
Source: PLoS One. 2015 Mar 24;10(3):e0120529. doi: 10.1371/journal.pone.0120529 (PMC4372558; doi:10.1371/journal.pone.0120529)
Supplement: S2 Protocol — (DOCX) [file pone.0120529.s005.docx]

**임상 연구계획서**

1. 연구 제목

진정 방법에 따른 내시경 점막하 박리술의 시술 성적 평가

1. 연구 필요성 및 개요

내시경 점막하 박리술은 소화기계 종양을 내시경적으로 일괄절제를 가능케 한 내시경 절제술의 한 방법이다. 전통적 방법인 내시경 점막 절제술과 비교할 때, 일괄절제가 어려운 크기 20 mm 이상의 병변에 대해서도 일괄절제를 가능하다는 점이 큰 차이점이다.[^1^](#_ENREF_1)^,^[^2^](#_ENREF_2) 내시경 점막하 박리술이 처음 소개된 이래로 치료 내시경 분야에서 기술 및 장비의 괄목한 발전이 이뤄지기는 하였으나, 여전히 내시경 점막하 박리술은 시간 소요가 많고 시술자의 기술이 중요한 시술 방법이다.[^2^](#_ENREF_2)^,^[^3^](#_ENREF_3) 따라서 일반적인 위내시경 혹은 다른 치료 내시경과 비교할 때 성공적인 진정 혹은 마취 여부가 내시경 점막하 박리술의 성공 여부에 있어 매우 중요하다.[^4^](#_ENREF_4) 아직까지 내시경 점막하 박리술 시행 시의 표준적인 진정 방법은 제시된 바가 없으나, 대부분 midazolam 이나 propofol 을 이용한 진정을 시행한다.[^3-7^](#_ENREF_3) 현재 진정 시에는 propofol을 기본으로 하는 경우가 많은데, 이는 다양한 내시경 시술의 진정 시 benzodiazepine 계열 약제에 비해 propofol 이 진정 유도와 회복에서 우수한 효과를 나타냄이 입증되었기 때문이다.[^8-14^](#_ENREF_8) 그리고, 두 전향적 연구에서 간헐적인 midazolam 주사 요법과 비교할 때 지속적인 propofol 주입 요법 역시 안전함이 입증되었다.[^4^](#_ENREF_4)^,^[^15^](#_ENREF_15) 하지만, propofol을 내시경 의사가 주입할 지 마취과 의사가 주입할 지에 대한 문제는 아직 논란이 있다. 미국 식약청에서는 propofol 은 전신 마취 수련을 받은 자에 의해서만이 투약될 수 있다고 허가한 반면,[^16^](#_ENREF_16) 미국 소화기협회에서는 적절하게만 수행될 수 있다면 소화기계 전문의가 propofol 을 이용하여 진정을 시행하는 것이 의료법적으로 타당하다고 규정하고 있기 때문이다.[^17^](#_ENREF_17) 그러나, 현재까지 propofol을 이용한 진정이 타당한가에 대해서는 법적, 안정성, 혹은 경제적 효용성에 대한 문제와만 연관되어 연구가 진행되었고, 진정 방법이 내시경 점막하 박리술의 시술 성적에 미치는 영향에 대해서는 연구가 이뤄진 바가 없다. 이에 본 연구진은 내시경 점막하 박리술 시술 성적에 진정 방법이 미치는 영향을 전향적으로 분석하여 내시경 점막하 박리술 시의 효율적인 진정 방법을 제시해 보고자 한다.

3. 연구 목적

내시경 점막하 박리술 시 진정 방법이 시술 성적에 영향을 미치는 지를 확인하고자 한다.

4. 연구 수행장소 및 기간

가. 수행장소: 연세대학교 의과대학 세브란스병원

나. 기간: IRB 승인일 이후 24개월

5. 피험자의 선정 또는 제외기준 및 스크리닝 검사 항목

가. 선정 기준

1) 만 20세 이상 80 세 미만의 남녀

2) 내시경 점막하 박리술이 가능한 조기위암 혹은 위선종

A. 조기위암

가) 크기와 상관없이 궤양을 동반하지 않고 분화도가 좋으며(well- to moderate-differentiated) 점막 내에 국한(T1a)된 것으로 판단되는 위암

나) 궤양 동반 여부와 상관없이 분화도가 좋으며 점막 내에 국한된 것으로 판단되는 크기 3 cm 이하의 위암

다) 분화도와 상관없이 궤양을 동반하지 않고 점막 내에 국한된 것으로 판단되는 크기 2 cm 이하의 위암

B. 위선종

가) 내시경적 육안 소견상 크기 2 cm 이상의 위선종

3) ECOG performance status 0 – 1

4) American Society of Anesthesiologist (ASA) Physical Status 1 – 3 (표 1)

5) 추적관찰하기에 적절한 환자 순응도를 보이고 지리적 거리가 적당한 환자

6) 치료시작 이전에 피험자 동의서에 서명을 하고 언제라도 불이익 없이 임상시험에서 중도에 탈락할 권리가 있음을 이해하고 있는 환자

나. 제외 기준

1) 이전에 부분 위절제술을 시행 받은 환자

2) 이전에 위루술을 시행 받은 환자

3) 이전에 내시경적 절제술을 시행했던 병변에 대해 내시경 점막하 박리술을 시행해야 하는 환자

4) 내시경 점막하 박리술을 시행할 병변이 세 개 이상인 환자

5) 다른 시술로 인해 ESD 전 24시간 이내에 진정을 시행 받은 환자

6) 임신 혹은 수유 중인 환자

7) 본 연구에 사용하는 약제에 allergy 과거력이 있는 경우 (달걀, 콩, sulfite 등)

8) 심각한 신경학적 또는 정신적 동반 질환이 있는 경우 (간질 또는 치매 등)

9) 환자 및 보호자의 고지된 동의 (informed consent)를 획득하지 못한 환자

다. 스크리닝 검사 항목

1) 위내시경 및 조직검사

2) 전신 장기 기능 상태 확인

A) 골수기능: 절대 호중구 수 ≥ 1,500 /mm3, 헤모글로빈 ≥ 9.0 g/dL, 혈소판수 ≥ 100,000 /mm3

B) 혈액응고기능: 프로트롬빈시간 < 1.5 INR, 활성부분트롬빈시간 ≤ 1.5 x 정상범주

C) 간기능: 혈청 총 빌리루빈 < 2.0 mg/dL, AST/ALT < 2.5 x 정상범주

D) 신장기능: 혈청 크레아티닌 ≤ 정상범주 혹은 CCr ≥ 60 mL/min (Cockcroft-Gault equation 이용)

E) 전해질: sodium, potassium, chloride 수치에 유의한 이상 없음

라. 탈락 기준

환자는 어떠한 이유로라도 아무 때나 시험을 철회할 권리를 가진다. 시험자는 환자와 전화나 방문을 통한 접촉으로 또는 환자를 책임지는 가족과 접촉하여 철회사유를 알아내도록 한다. 시험철회가 이상반응이나 실험실 검사결과의 비정상으로 인한 것이라면 이에 대한 주요반응이나 검사결과가 CRF에 기록된다.

6. 목표 피험자의 수 및 산출 근거

1. 총 피험자수: 157명
2. 산출 근거

*H*_0_ : intermittent midazolam/propofol injection controlled by endoscopist (IMIE) 군의 시술자의 만족도 = continuous propofol infusion with opioid administration controlled by anesthesiologist (CPIA) 군의 시술자의 만족도

*H*_1_ : IMIE 군의 시술자의 만족도 ≠ CPIA 군의 시술자의 만족도

표본수는 예비시험 결과를 이용하여 산출함

IMIE군: 만족 6명, 보통 3명, 불만족 1명

CPIA군: 만족 8명, 보통 1명, 불만족 1명

Estimated effect size: 0.25 (자유도 2, 카이제곱 검정)

유의수준: 0.05, 검정력: 80%

중도탈락율: 1%

표본수: 157

7. 연구 설계 및 방법

가. 연구 설계의 개요

1) 본 연구는 단일기관, 공개, 무작위, 비교 임상시험으로 디자인 되었으며, 위암 혹은 위선종 환자 중 내시경 점막하 박리술을 시행하는 환자를 대상으로 한다.

2) 피험자가 임상시험에 참여할 것을 서면으로 동의하면, 임상시험계획서에 따라 필요한 검진 및 검사를 실시한 후 피험자 적합성 평가결과, 선정기준에 적합한 피험자에 한하여 임상시험을 진행한다.

3) 환자군 배정 방법

A) 층화 무작위 배정(stratified randomization)을 시행하며 층화에 사용하는 변수로는 조직학형 (내시경 점막하 박리술 이전에 시행한 조직검사에 따른 암 혹은 선종) 한 가지로 한다.

B) 각 층 별로 구역화 무작위 배정(blocked randomization)을 시행하여 IMIE 군 혹은 CPIA 군에 배정한다.

4) 진정방법

A) IMIE 군

가) 내시경 의사에 의해 진정을 시행하는 군으로 bolus 방식의 주입이 가능한 meperidine (pethdine®, Jeil Pharmaceutical Co. Lt., Daegu, Korea), midazolam (Midazolam®, Bukwang Pharm. Co. Ltd., Seoul, Korea), propofol(Pofol®, Dongkook Pharm. Co. Ltd., Seoul, Korea) 을 사용하여 진정을 시행한다.

나) 먼저, meperidine 50 mg 을 bolus 방식으로 근육 주사한다.

다) meperidine과 함께 체중 1 kg 당 0.05 mg의 midazolam 을 bolus 방식으로 정맥 주사한다.

라) 환자가 움직이거나 불편해하는 것이 관찰될 경우 Modified Observer Assessment of Alertness/Sedation (MOAAS) scale (표 2)을 체크하여 5~6점일 경우 진정이 불충분한 것으로 보고 propofol 0.25 mg/kg 을 정맥 주사하며, MOAAS scale 이 3~4점일 경우 환자가 통증에 반응하는 것으로 평가하여 meperidine 12.5 mg을 bolus 방식으로 정맥 주사한다.

B) CPIA 군

가) 마취과 의사에 의해 진정을 시행하는 군으로 infusion 방식의 주입이 가능한 remifentanil (Ulitiva®, GlaxoSmithKline, Co. Ltd., Genval, Belgium) 및 propofol 을 사용하여 진정을 시행한다.

나) 먼저, remifentanil 0.05 μg/kg 을 bolus 방식으로 정맥 주사한 후 0.08 μg/kg/min 의 속도로 infusion 방식으로 정맥 주사한다.

다) 이후, propofol 0.5 mg/kg 을 bolus 방식으로 정맥 주사한 후 2 mg/kg/h 의 속도로 infusion 방식으로 정맥 주사한다.

라) 환자가 움직이거나 불편해하는 것이 관찰될 경우 MOAAS scale을 체크하여 5~6점일 경우 진정이 불충분한 것으로 보고 propofol 을 0.25 mg/kg bolus 방식으로 정맥 주사한 후 propofol 의 주입 속도를 0.5 mg/kg/h 씩 단계별로 올린다.

마) MOAAS scale 이 3~4점일 경우 환자가 통증에 반응하는 것으로 평가하여 remifentanil의 주입 속도를 0.02 μg/kg/min 씩 단계별로 올린다.

바) 수축기혈압 90 mmHg 미만, 기저에서 20% 이상 감소, 혹은 SpO_2_ 90% 미만 소견이 관찰될 경우 propofol 의 주입 속도는 0.5 mg/kg/h 씩 단계별로 감량한다.

5) 진정 수준 및 활력 징후의 평가

A) 양 군의 목표 진정 수준은 모두 MOAA/S score 3 ~ 4 로 한다.

B) MOAA/S score 평가

1) 내시경 삽입 전

2) 내시경 삽입 후, 절개 전

3) 절개 후, 박리 종료 전

4) 박리 종료 시

5) 진정이 충분치 않거나 환자가 불편감이나 통증에 반응할 경우

C) 활력증후 측정: 매 5분마다

D) 시술을 방해하는 사건

1) Belching

2) Vomiting

3) Spontaneous moving

4) Physical restraint

E) 호흡 관련 사건

1) Chin lift

2) Increased O_2_ flow

3) Assisted mask ventilation

4) Intubation

8. 관찰항목

가. 환자요인

1) 연령

2) 성별

3) 흡연력

4) 과거력

5) ASA Class

6) 항혈소판제 혹은 항응고제 복용 여부

나. 종양요인

1) 조직학형 (암, 선종)

2) 육안적 모양 (elevated, flat, depressed)

3) 병변 위치

A) Upper third (fundus, cardia, upper body)

B) Middle third (mid body, lower body)

C) Lower third (angle, antrum, pylorus)

4) 병변 크기

5) 궤양 동반 여부

라. 시술요인

1) 일괄절제 여부

2) 완전절제 여부

3) 치료적절제 여부

4) 시술시간

5) 합병증 여부 (출혈, 천공, 흡인성 폐렴)

6) 진정 수준

9. 유효성 평가기준

가. 일차 목적: 진정 방법에 따라 시술자 만족도가 차이를 보이는지 분석한다.

나. 이차 목적

1) 진정 방법에 따라 ESD 성적이 차이를 보이는지 분석한다.

2) 진정 방법에 따라 환자 만족도가 차이를 보이는지 분석한다.

다. 통계분석방법

1) 양 군에 무작위로 배정된 모든 환자는 intention-to-treat (ITT) 원칙을 적용하여 분석한다.

2) 일차 목적의 통계 분석으로는 카이제곱 검정 혹은 피셔의 정확한 검정을 이용한다.

3) 이차 목적의 통계 분석으로는 카이제곱 검정 혹은 피셔의 정확한 검정을 이용한다.

4) 중간 분석은 시행하지 않는다.

5) 결측치가 발생한 경우, complete case analysis 를 시행한다.

10. 연구 윤리 및 규제

가. 본 연구는 The ethical guidelines of the 1975 Helsinki Declaration 과 International Conference on Harmonisation of Technical Requirements of Pharamceuticals for Human Use (ICH) Note for Guidance on Good Clinical Practice (ICH, Topic E6, 1995) 을 따른다.

나. 본 연구는 세브란스병원 연구윤리심의위원회에서 승인되었다.

다. 보상

본 연구에서 사용되는 두 가지 진정 방법은 모두 현재 임상에서 널리 사용되는 진정 방법으로서, 본 연구 참여와 관련한 금전적인 보상은 없다.

11. 참고 문헌

1. Miyamoto S, Muto M, Hamamoto Y, et al. A new technique for endoscopic mucosal resection with an insulated-tip electrosurgical knife improves the completeness of resection of intramucosal gastric neoplasms. Gastrointest Endosc 2002;55:576-81.

2. Gotoda T, Yamamoto H, Soetikno RM. Endoscopic submucosal dissection of early gastric cancer. J Gastroenterol 2006;41:929-42.

3. Akasaka T, Nishida T, Tsutsui S, et al. Short-term outcomes of endoscopic submucosal dissection (ESD) for early gastric neoplasm: multicenter survey by osaka university ESD study group. Dig Endosc 2011;23:73-7.

4. Yamagata T, Hirasawa D, Fujita N, et al. Efficacy of propofol sedation for endoscopic submucosal dissection (ESD): assessment with prospective data collection. Internal medicine 2011;50:1455-60.

5. Imagawa A, Fujiki S, Kawahara Y, et al. Satisfaction with bispectral index monitoring of propofol-mediated sedation during endoscopic submucosal dissection: a prospective, randomized study. Endoscopy 2008;40:905-9.

6. Lee H, Yun WK, Min BH, et al. A feasibility study on the expanded indication for endoscopic submucosal dissection of early gastric cancer. Surg Endosc 2011;25:1985-93.

7. Ahn JY, Jung HY, Choi KD, et al. Endoscopic and oncologic outcomes after endoscopic resection for early gastric cancer: 1370 cases of absolute and extended indications. Gastrointest Endosc 2011;74:485-93.

8. Heuss LT, Froehlich F, Beglinger C. Changing patterns of sedation and monitoring practice during endoscopy: results of a nationwide survey in Switzerland. Endoscopy 2005;37:161-6.

9. Cohen LB, Wecsler JS, Gaetano JN, et al. Endoscopic sedation in the United States: results from a nationwide survey. The American journal of gastroenterology 2006;101:967-74.

10. Benson A, Cohen LB, Waye JD, et al. Endoscopic sedation in developing and developed countries. Gut and liver 2008;2:105-12.

11. Horiuchi A, Nakayama Y, Hidaka N, et al. Low-dose propofol sedation for diagnostic esophagogastroduodenoscopy: results in 10,662 adults. The American journal of gastroenterology 2009;104:1650-5.

12. Cote GA, Hovis RM, Ansstas MA, et al. Incidence of sedation-related complications with propofol use during advanced endoscopic procedures. Clin Gastroenterol Hepatol 2010;8:137-42.

13. Riphaus A, Rabofski M, Wehrmann T. Endoscopic sedation and monitoring practice in Germany: results from the first nationwide survey. Zeitschrift für Gastroenterologie 2010;48:392-7.

14. Bo L, Bai Y, Bian J, et al. Propofol vs traditional sedative agents for endoscopic retrograde cholangiopancreatography: a meta-analysis. World Journal of Gastroenterology 2011;17:3538-43.

15. Kiriyama S, Gotoda T, Sano H, et al. Safe and effective sedation in endoscopic submucosal dissection for early gastric cancer: a randomized comparison between propofol continuous infusion and intermittent midazolam injection. Journal of gastroenterology 2010;45:831-7.

16. Aisenberg J, Cohen LB, Piorkowski JD. Propofol use under the direction of trained gastroenterologists: an analysis of the medicolegal implications. The American journal of gastroenterology 2007;102:707-13.

17. Matsui N, Akahoshi K, Nakamura K, et al. Endoscopic submucosal dissection for removal of superficial gastrointestinal neoplasms: A technical review. World J Gastrointest Endosc 2012;4:123-36.

18. Cohen LB, Delegge MH, Aisenberg J, et al. AGA Institute review of endoscopic sedation. Gastroenterology 2007;133:675-701.

표 1. American Society of Anesthesiologist Physical Status Classification System

| ASA Physical Status 1 | A normal healthy patient |
| --- | --- |
| ASA Physical Status 2 | A patient with mild systemic disease  Ex) Well controlled hypertension or DM, pregnancy, old tuberculosis, mild obesity (BMI>25), smoker (without COPD) |
| ASA Physical Status 3 | A patient with severe systemic disease  Ex) 2 or more ASA physical status 2, poorly controlled hypertension or DM, arrhythmia, well-controlled congestive heart failure, stable angina, old heart attack, obesity (BMI>35), chronic renal failure, COPD, cerebral vascular attack, active tuberculosis |
| ASA Physical Status 4 | A patient with severe systemic disease that is a constant threat to life  Ex) Unstable angina, symptomatic COPD, symptomatic congestive heart failure, hepatorenal failure, ESRD, stable sepsis |
| ASA Physical Status 5 | A moribund patient who is not expected to survive without the operation  Ex) multi-organ failure, unstable sepsis, hypothermia, severe coagulopathy |
| ASA Physical Status 6 | A declared brain-dead patient whose organs are being removed for donor purposes |

표 2. Modified Observer Assessment of Alertness/Sedation Scale[^18^](#_ENREF_18)

| Responsiveness | Score |
| --- | --- |
| Agitated | 6 |
| Responds readily to name spoken in normal tone (alert) | 5 |
| Lethargic response to name spoken in normal tone | 4 |
| Responds only after name is called loudly and/or repeatedly | 3 |
| Responds only after mild prodding or shaking | 2 |
| Does not respond to mild prodding or shaking | 1 |
| Does not respond to deep stimulus | 0 |
